# American Academy of Dental Science

**Published:** 1902-11

**Authors:** 

**Affiliations:** American Academy of Dental Science


### AMERICAN ACADEMY OF DENTAL SCIENCE.

THE regular monthly meeting of the Academy was held at Young's Hotel, Boston, on Wednesday evening, May 7, 1902, at six o'clock, President Bradley in the chair.

The minutes of the previous meeting were read and approved.

*President Bradley.*—This evening we are to have the pleasure of listening to a paper entitled "Amputation of the Palatal Roots of Superior First Molars for the Prevention of Antral Abscesses," by a gentleman who was associated with Dr. Harrison, of Philadelphia, some time ago, and is now a resident and in practice in this city. I have the pleasure of introducing to you Dr. Isidore Lett, D.D.S., of Boston.

(For Dr. Lett's paper, see page 784.)

### DISCUSSION.

*President Bradley.*—The discussion of the paper we have listened to is to be opened by a guest of the Academy. I have the pleasure of introducing Dr. Timothy J. Reardon.

*Dr. Reardon.*—The item interests me from another stand-point than a dental one, in this way, that I, being a nose man, look at it from that end of the science. I was interested in the doctor's paper because he suggested a method of treating the antrum which, to my mind, may be practical, instead of sacrificing the teeth, as is done to-day by the surgeon; or, at least, as I do. I always recommend, first, the taking out of a molar, for the simple surgical reason that the drainage is the best. I thought as he read his paper that perhaps if the palatal root was large enough, and could be removed surgically by a dentist, it would answer the purpose and save the tooth. As I take it, a dentist will do about anything to save a tooth; or, at least, he should; but a surgeon has no hesitancy about taking it out, or ordering it out.

Now, about diagnosis of the antrum. It is very well to probe through the dental foramen into the antrum experimentally, in treating a tooth; but still I feel that there are other methods of finding out, and perhaps they should be used. It is a very simple matter to pass a canula from the floor of the nose into the antrum, and I think it should be done. There is practically no risk about it; there are very simple rules about it, and anybody could carry it out.

As to the hard cases of antrum disease,—*i.e.*, those that will hang along for a year or more,—undoubtedly in a great many of these antrum cases that last so long there is some bone disease. Perhaps the entire wall, as in osteomyelitis acuta, may be carious, and it has been found that necrosis of the outer wall of the nose can exist, as in syphilis and tuberculosis; so there is no question that trouble in the other parts can exist without being especially connected with the teeth. It is surprising how often antrum diseases exist. There is hardly a death from infectious disease where pus is not found in the frontal sinus, and generally in the antrum, for the simple reason that the antrum is so placed with its natural opening that the frontal sinus empties practically into it, or in the great majority of cases it does that. If you find an antrum, it may be simply a receiver for the pus from these other cavities. Again, if the cavity is not well ventilated, healing will be retarded.

Tumors of the antrum are quite rare. Many of them are œdematous granulations. A tooth will be carious, and the bone will give out these large excrescences, sometimes as large as a cherry, and originating in a diseased tooth.

That case mentioned by the doctor suggested to me that this palatal root may present conditions that it would be well to study. Men interested in it could carry on special investigations which might be valuable.

Another thing suggested to me was the passing of this probe through the root into the antrum. It was related to me by a very distinguished man, with whom I studied abroad, that a lady was accountable for the discovery of the antrum. She was using a gold toothpick, which disappeared, and Highmore, observing it, began to study this cavity. Probably he did not give us a great deal of knowledge about it, but apparently he was the first man to investigate it thoroughly.

I think at one time most antra were thought to be of tooth origin, but I think that is a very erroneous idea. Perhaps thirty per cent. of them are. During the influenza epidemic ten years ago we knew nothing of the ethmoidal and sphenoidal sinuses, except that the eye men here and there found diseases of them. But we do not wait to-day for sinus diseases to rupture externally.

Another thing which I would like to talk to you about, is neuralgia. We have a great deal to do with it. It is often caused by the teeth. I am pleased to say to you that dentists hesitate a great deal to-day about operating and removing teeth for tic douloureux. Very often these teeth are absolutely sound. I have seen cases myself where there is some mysterious cause for the pain; I have seen, within the past few weeks or months, a few cases. I was told one day by a nerve man, while riding on a car, that he treated one of these cases with iodide of potash, and it got well. I saw two cases: to one man I gave iodide of potash, and it cleared up. This man also had Rigg's disease of the teeth, but they had never been removed. He gave a history of syphilis. The other case was also syphilitic, and he responded to the same treatment.

These things should be thought of before any action is carried out in regard to removing teeth. The medical profession is against it to-day as much as the dental profession was ten years ago. I think the advanced men hesitate always about removing teeth. Other causes may have been looked for, but it is still a mystery in many cases to-day as much as ever.

I do not believe, gentlemen, I have anything more to say to you, except one thing, that has lately been observed, and that is in relation to peritonsillar abscesses caused by a carious molar tooth, principally of the upper jaw. Several cases have been reported. I have never seen one myself, but henceforth I shall look for it. I always seek for carious teeth in any suppurative condition of the nose. I believe there is some connection between diseases of the mucous membrane of the nose and the teeth. Just how the two are connected I cannot state, but it exists, and the combination is so frequent that it has got most of the nose and throat men to thinking that there is some connection. I dare say, if you will watch, you will find hardly a child that during the eruptive stage of the teeth does not have a good deal of suppurative or catarrhal condition of the nose. I usually pass it by, and the chances are it will clear up very shortly, and it generally does.

I think that is all I have to say to you, gentlemen, on that subject.

*President Bradley.*—The subject is now open for general discussion.

*Dr. Fillebrown.*—The excellent paper of the essayist brought to my mind the amputation of the palatal root of a molar to relieve an abscess and save the tooth. It was the year I attended the Harvard Dental School, 1869. Dr. Keep was then dean. One day he showed two or three of us in his office (by the way, we occupied his office quite as much as we did the Dental School building) a molar tooth in his own mouth with the palatal root amputated. It was done by his brother quite a number of years before, and the tooth had remained strong and good. Not many years after that he died, and I presume the tooth remained good unto the end.

It interested me very much then; consequently, I have repeated the operation in a number of instances, and always with success. Dr. Keep spoke of other cases in his practice, but how generally it has been practised I do not know. We have not much literature on the subject, either because it has not often been done, or because it was considered so simple and reasonable that it has not been discussed. It certainly is a very desirable operation.

I remember a case of necrosis in Dr. Keep's clinic that was quite interesting. A young girl, about ten years old, had necrosis around a bicuspid tooth. It was attributed to the application of arsenic. She came in one day when the sequestrum was just ready to be removed. The old doctor thought he could help her to be relieved of the pain. I remember he asked her to breathe deeply, had her repeat it several times, and then he said, "Just hold your breath a minute;" and while she held her breath he passed an excavator up and drew out the sequestrum, and the patient did not flinch. That gave me an inkling of what has since developed into a fine way to overcome the pain and dread of slight operations.

Dr. Reardon spoke of the direction of the infundibulum and its form from the frontal sinus to the antrum. I think it will be found that I was the first one that had found out or had mentioned that fact in the matter. Many others, Dr. Cryer among them, had found out that they could go into the antrum through a hole in the alveolus and reach across it, and then pass up and reach into the frontal sinus. That is a good deal like being able to put a pole into the window of a room and pass it out through the opposite door. But the fact that the infundibulum is a trough with a wall that rises up high on the nasal surface and conducts all the secretions from the frontal sinus down into the antrum, was, I think, not recognized before.

He makes a good recommendation to be careful about condemning the antrum too soon, for we may find that it is the frontal sinus that is at fault. A friend of mine sent a patient to me one day, with evident trouble in the antrum. Upon investigation I found it to be the frontal sinus. Opening that, I found it filled with a jelly-like substance. Proper treatment of the sinus soon removed the secretions and relieved the antrum.

There are some forms of antrum trouble which I think are better relieved by opening through the fossa. If you have any extended morbid growth in the antrum that you need to remove, it is rather hard doing it through the alveolus, although generally I have been able to do it that way, and have done so in most of the cases I have had.

I remember one case that came to us in the clinic at the Harvard Dental School, showing the persistence of trouble with the antrum; and also emphasizing the fact that Dr. Reardon alluded to, that trouble is not always confined to the region of the tooth, even if it has its origin in an abscess on the tooth. This patient had necrosis, including the alveolar walls of the upper incisors and the left cuspid and bicuspid. The teeth and sequestrum were removed, which left, of course, a good opening into the antrum. The case bothered me for nearly a year before I could get the antrum healed. In the most distal portion of the antrum persisted a little spot which secreted pus for a long time, and it required many applications of strong carbolic acid before it would heal up. I diagnosed the condition by winding a probe with cotton, and gradually found the angle needed to reach the spot without friction on the sides. Then I would carry it into the place and hold it for a moment, remove it carefully, and by noting the spot on the cotton I would be able to locate the spot, and about its size. I remember it persisted at about the sixteenth of an inch across for a long time.

Necrosis does not enter very largely into the discussion to-night, but a case that appeared at the clinic last Friday is quite interesting and instructive. It had this bearing; it enforces the necessity of more accurate knowledge of the possibilities of dental practice and a more keen recognition of the limits of dental service and of dental preservation. This woman came into the clinic with six or seven front teeth so loose that she had to tie a string around them to hold them in. She was sent there by a neighboring operator, who did not wish to assume the responsibility of a decision as to what to do. There was extensive necrosis which was seriously endangering the patient's health. I removed the teeth at once. The sequestrum had not separated, so I passed my forceps around and nipped off all the roughened alveolus. This should have been done before. I just wished to emphasize the fact that we as dentists oftentimes try to do too much.

There was another case in the clinic last Friday, sent through the advice of a mutual friend, where the upper third right molar had been removed some months before. She had had trouble in that region for some time previous to the extraction, and the trouble still persisted; pus and blood kept dropping away from the socket. It had been treated over and over again, and yet had not got well. I could not feel anything out of the way, but the second molar looked dark. I found it was filled with a cotton dressing. It had been treated for a long while. I also found by a casual remark that repeated attempts had been made to stop up the roots, and every time swelling and pain had ensued. I advised its extraction, for I believe that second molar was the cause of the trouble. I have learned since that it was extracted, with the result of finding that all three of the roots were abscessed and considerably absorbed. Of course, one of those abscesses had been discharging through the seat of the third molar all this while. The other abscess had not opened externally, and consequently when the attempt was made to plug it up it made the trouble. There was a case where it was a great mistake to save the tooth so long. It was due to the patient, to the science of dentistry, and the sensible application of it that it should have been removed long before.

You remember, a few years ago, Dr. Sexton, of New York, wrote an article and published it, in which he took the ground that too many dead teeth were saved. Many cases of ear trouble were caused and continued by the presence of these dead teeth. I remember Dr. Frank Abbott and others took up the cudgel, and Dr. Sexton was used rather roughly and with considerable ridicule. I am satisfied that Dr. Sexton was right, and that to-day there are teeth being kept in the mouths when in all reason and righteousness, so far as the patient's interests and the interests of the science of dentistry are concerned, they ought to be removed.

*Dr. Drexel.*—I want to endorse, from my experience, the essayist's remarks about the use of hydrogen dioxide. I do not believe it has the healing quality about it. I do not believe it should be used in the antrum, or even in indolent ulcers. I think it retards healing.

I am a worshipper of the salts of silver. I have had beautiful results from its use, and in it we have something that is bland, no violent action, and the mucous membranes take kindly to it. It can be used in pretty strong solution; and, in fact, in ulcers can be dusted on, without getting up any action, such as carbolic acid produces, for instance. I think salts of silver will bear your investigation.

*President Bradley.*—We should be very glad to hear from any one on this subject.

*Dr. George T. Baker.*—I have just one thought I want to express, in regard to the saving of teeth, and that is, we have all noticed that in a case of alveolar abscess where there is no fistula, it is sometimes very difficult to effect a cure, but where there is one, it is usually very much easier done. Where there is no fistula, I have found it a great help to make one through the gum. And in making one of that kind, I have found that a drill which seems to work better than any other is a perfectly smooth drill. I have tried it with an ordinary round bur, and have found that the bur will catch in the tissues and give considerable trouble; so that a perfectly smooth drill, like this that I have, is better. These have been used a good many times, and they are worn down. They ought to be a little longer; they were longer originally. They are perfectly smooth, just an ordinary drill; one is for the right-angle hand-piece. I will pass them around.

*Dr. Williams.*—An eminent surgeon once remarked at one of our meetings that it was formerly thought that the easiest way to dispose of any obstacle was to throw it away, as Dr. Reardon intimated is too often done in dental surgery. It is the simplest way to dispose of a thing. But I think a little more thought of preservation of a tooth, if it is valuable, a little more preservation and knowledge of the ways of correcting the ailments of the roots of the teeth, might save a great many roots and teeth. This one method of amputation, for instance, gives good results in many cases by proper treatment. The question should be decided, I think, somewhat in relation to the value of the tooth; if it is valuable, every effort should be made to save it.

*Dr. Fillebrown.*—I do not wish to be misunderstood. I would not sacrifice a tooth until every means to save it had been exhausted. But after a while, as in this case of last Friday, where the trouble had continued for months, and there was no prospect of making it any better, then I say it is not right to keep it there, to the injury of the patient's health.

I wish to ask Dr. Reardon whether these cases of sympathy between the teeth and the nose were between the back teeth—*i.e.*, the molar teeth—or the bicuspids and the six anterior teeth? Abscesses from the six anterior teeth, or from the bicuspid teeth, especially the first, very seldom, I think, interfere with the antrum.

I know I was treated with a little derision here in this Academy one night some years ago, when a gentleman had a case of abscess on the lateral incisor, or central incisor, and could pass a probe, he said, for an inch or two into the antrum. I raised the question that I thought the probe did not get into the antrum, and it caused a smile from him and several others. Now, gentlemen, I am sure to-night that in those cases it will not ordinarily go into the antrum, but the abscess will burrow through back between the plates of the palate bones, pressing both ways, and form a cavity as big as some antra. I know that very evening I cited a case that I had where I had passed a probe in the length of my little finger, and yet I was sure then, and am sure to-day, that it did not go into the antrum at all. The nervous relation between the six, eight, or ten anterior teeth and the floor of the cavities of the nose is very close, and it is not strange that troubles on those teeth should affect them. What I want to know is, whether Dr. Reardon has been able to distinguish between the molar and the anterior teeth?

*Dr. Reardon.*—I will state that in the last case I saw in which there was no antrum it was the molars, and chiefly the second molars, on both sides that were affected.

And in connection with this separation of the nose, I cannot state (because we have not got that far) whether it is due to absorption through the lymphatics, or due to some disturbance in connection with the nerves.

Here is a case I took a note of a short time ago. (Reads note.) It does not give just what teeth were involved, and that is one of the dangers of taking short notes from editorials,—*i.e.*, to be accurate. If I ever wrote up the subject, I would look up these cases and find just what teeth were involved. It just shows that there are a lot of things that need investigating which are on the border line of dentistry and rhinology.

*Dr. Fillebrown.*—That brings a case to my mind that I had within a few weeks, which is so much to the point that I must take the time to refer to it. A gentleman had had trouble with a right superior cuspid, on which there had been an abscess for a long time, and the dentists were all trying to save it, and for months, yes, more than that, for two or three years, they had been keeping it along. The minute they closed it up, pain would follow. Finally, they took the tooth out, and it was apparently well for quite awhile. Presently he began to blow pus from his nose, and that went on for four or five months. Two or three times he had the antrum opened, supposing the trouble was there, and they found no trouble whatever. The trouble was as I diagnosed it in the beginning, the remnants of that old abscess on the right superior cuspid tooth, and it had remained there and worked through and was discharging into the nose. I treated it on that theory, keeping the thing open until it healed, and it proved the cause of the trouble. So that oftentimes when there is pus occurring through the nose, we ought to look out and consider whether it is one of the anterior teeth that may be in trouble, or whether it is the nose itself.

*Dr. Reardon.*—I would like to state that in diseased conditions of the nose it has been my practice to tampon with a strip of gauze, generally soaked in peroxide of hydrogen. Invariably I find that if any of the teeth are filled, and generally, so far as I have been able to observe, even molars, they will ache if they are in that condition. That has been my observation. I could look it up in my notes and give more definite information.

*Dr. Werner.*—Speaking of the abnormal condition of the antrum, we must also take into consideration the abnormal condition of the roots in that locality. Yesterday I had a case where good judgment dictated the extraction of the right superior second molar, which had no buccal and no palatal roots, but simply the average root of a very small normal bicuspid. How ridiculous it would have been to attempt to amputate roots that did not exist, though appearances in the mouth indicated distinct palatal and buccal roots.

*Dr. Brackett.*—In connection with this minor surgery of the teeth I may mention something which I learned a few months ago from Dr. George H. Ames. It is a method of giving escape to the products of inflammation about the apex of a closed root, and this in advance of any perforation which nature could make, thus very much shortening the period of suffering. Usually by percussion and pressure in different directions we may determine which particular root is the seat of a developing abscess. The procedures are, first, to benumb with cocaine the gum overlying the affected root; secondly, to plunge through this gum over the apex a small round burnisher which has been heated so as to make actual cautery; thirdly, with an engine drill or bur to perforate the alveolar wall. The cocaine makes the operation almost painless. Unlike a lance cut, the burned perforation in the gum is followed by no hemorrhage. You see the bare process at the bottom of the opening in the gum, and are able to direct by sight the action of the drill.

This method may be one of the resources in the small class of cases in which pericemental troubles develop with teeth whose pulps have been removed and canals and cavities filled with materials which it is not feasible to remove. In the one case in which I have followed this method, the tooth, notwithstanding unusual complications, has been saved, and is now doing comfortable service. The instrument with which the gum is burned should be a small one; otherwise there is after-suffering from the burn, and the repair is too tedious. The closing of the gum any way after the burn is likely to be even slower than is the case when an alveolar abscess is opened with the engine gum trephine suggested years ago by Dr. William H. Rollins.

*President Bradley.*—If there is no further discussion upon the subject, Dr. Lett will close the discussion.

*Dr. Lett.*—I should like to first answer Dr. Reardon's argument in regard to draining an abscess which would empty into the antrum, ventilating that through the alveolus, followed by the extraction of the palatal root. In the majority of those cases which I have seen there is more or less of the carious degeneration of bone. In most cases, when that is removed, there has been so much taken away that it would leave very little foundation for the teeth, and I have always advocated extraction, for fear of the recurrence of this trouble.

In regard to the dioxide, I should like to say that whenever I use it in cases of cleaning out parts surrounding an abscessed root, or anything similar, I add about ten grains of bicarbonate of soda and make a saturated solution in water, and add about half an ounce of the dioxide; and I find that that overcomes the unpleasant and caustic effect of the dioxide.

In regard to making fistulas for treating teeth, or draining abscesses at the roots of those teeth which will not succumb to ordinary treatment, I use the same method that Dr. Baker has described, except that I use the spear drill that White has in stock; and I have been very successful; probably not as successful as he has been, but it has answered all my purposes. It is an ordinary spear-pointed drill.

In regard to this subject of tic douloureux, I remember a very violent case of a man's attempting suicide on account of the excruciating pain, for which I tried phosphorus, one-twelfth grain doses, watching the patient very closely, and tincture of chloride of iron. This treatment cured the patient.

In regard to upper tonsillar abscesses, I have met quite a few cases, and in my experience most of them have been caused by impacted wisdom-teeth; and a common point of diagnosis between an abscess in the tonsil as a direct infection in the tonsil gland itself, or from a wisdom-tooth, is that when the tonsil is not involved with any dental trouble, the patient can open and close his mouth freely, with little or no pain; but where it results from an impacted wisdom-tooth, there is more or less ankylosis of the jaw.

I have a case in direct antipathy to Dr. Fillebrown, in regard to an abscess in the frontal sinus. I saw a case in consultation with a physician a short time ago, in which this patient complained of severe pain from neuralgia, and also pain in the region of the frontal sinus. From the right eye there was an overflow of tears.

I do not know whether that was caused by the closing of the nasal duct, or from over-stimulation of the lachrymal gland. I found the right first molar loose. Perhaps I should not have known there was any trouble there, but I passed a probe through the tissues in between the roots, and I felt satisfied that it was an abscess of the antrum; of course, there being an indirect connection between the frontal sinus and the antrum, very often found there when there is an abscess in the frontal sinus. It was a delicate question, because this doctor had sent this case in, and he pronounced it an abscess in the frontal sinus, and with no complications. I felt sure that if the tooth was extracted the patient would get relief. So he went back to this dentist and compelled him to remove the tooth, and immediately there was a flow of pus, and the unpleasant symptoms disappeared.

*Dr. Werner.*—Second molar?

*Dr. Lett.*—First molar. I was looking over some notes to-day, and I came across a case which Dr. J. D. White, of Philadelphia, treated, which was published in the *Dental Cosmos,* in regard to antral abscesses, or, at least, pus in the antral cavity caused by the buccal roots of a second molar. The tooth was explored, and the bulbous portion of the pulp and the buccal filaments were dead, but the filaments in the palatal root were still alive. An application of arsenic was made in that, and the filaments successfully removed, without any pain. Dr. White then extracted the tooth and found that there was no opening between the palatal root and the antrum, but through the buccal alveolus he passed into the antrum. Whether that was caused by an abscess on the end of this root, and burrowing through the alveolar process into the antrum, or whether, as he thought, infection resulted from coming in contact with the pus from the nose of a pet horse which he went to see frequently, he does not state very definitely, but he makes the suggestion that an infection came from this pus to the antrum.

In regard to saving teeth, I saw a very interesting case, about seven years ago, of a woman being brought into the hospital with a fracture of the superior maxillary bones. There was a quantity of pus there, and Dr. M. H. Cryer made an effort to save this by opening freely and draining out the pus, and working the parts and together wiring them in place, which resulted very successfully. He scraped off the edges of the bone, and had very good success with the case, which was discharged in about three weeks.

*President Bradley.*—Is there anything further to come before the Academy? I am sure that I express the feeling of every fellow of the Academy when I say the thanks of the Academy are due to Dr. Lett and Dr. Reardon for the paper and the discussion. All those who are in favor of expressing this same sentiment will manifest it by saying, "Ay."

Voted.

Adjourned until October meeting.

CHARLES H. TAFT, D.M.D.,
*Editor American Academy of Dental Science.*

